# Fentanyl-Induced Respiratory Depression and Locomotor Hyperactivity Are Mediated by μ-Opioid Receptors Expressed in Somatostatin-Negative Neurons

**DOI:** 10.1523/ENEURO.0035-23.2023

**Published:** 2023-06-27

**Authors:** Andreea Furdui, Carolina da Silveira Scarpellini, Gaspard Montandon

**Affiliations:** 1 Keenan Research Centre for Biomedical Science, St. Michael’s Hospital, Unity Health Toronto, Toronto, Ontario M5B 1W8, Canada; 2 Institute of Medical Science, Faculty of Medicine, University of Toronto, Toronto, Ontario M5S 1A8, Canada; 3 Division of Respirology, Department of Medicine, University of Toronto, Toronto, Ontario M5S 3H2, Canada

**Keywords:** brainstem, breathing, fentanyl, opioid, respiration, somatostatin

## Abstract

Opioid drugs are widely used as analgesics but cause respiratory depression, a potentially lethal side effect with overdose, by acting on μ-opioid receptors (MORs) expressed in brainstem regions involved in the control of breathing. Although many brainstem regions have been shown to regulate opioid-induced respiratory depression, the types of neurons involved have not been identified. Somatostatin is a major neuropeptide found in brainstem circuits regulating breathing, but it is unknown whether somatostatin-expressing circuits regulate respiratory depression by opioids. We examined the coexpression of *Sst* (gene encoding somatostatin) and *Oprm1* (gene encoding MORs) mRNAs in brainstem regions involved in respiratory depression. Interestingly, *Oprm1* mRNA expression was found in the majority (>50%) of *Sst*-expressing cells in the preBötzinger Complex, the nucleus tractus solitarius, the nucleus ambiguus, and the Kölliker–Fuse nucleus. We then compared respiratory responses to fentanyl between wild-type and *Oprm1* full knock-out mice and found that the lack of MORs prevented respiratory rate depression from occurring. Next, using transgenic knock-out mice lacking functional MORs specifically in *Sst*-expressing cells, we compared respiratory responses to fentanyl between control and the conditional knock-out mice. We found that respiratory rate depression by fentanyl was preserved when MORs were deleted only in *Sst*-expressing cells. Our results show that despite coexpression of *Sst* and *Oprm1* in respiratory circuits and the importance of somatostatin-expressing cells in the regulation of breathing, these cells do not mediate opioid-induced respiratory rate depression. Instead, MORs found in respiratory cell populations other than *Sst*-expressing cells likely contribute to the respiratory effects of fentanyl.

## Significance Statement

Opioid drugs cause respiratory depression, a potentially lethal side effect with overdose, by acting on μ-opioid receptors in brainstem regions regulating breathing, therefore limiting their effective use as analgesics. Somatostatin is a major neuropeptide found within these brainstem circuits, but it is unknown whether somatostatin circuits regulate respiratory depression by opioids. We found that somatostatin-expressing neurons coexpress μ-opioid receptors in respiratory circuits but that respiratory rate depression by fentanyl was preserved despite genetic deletion of μ-opioid receptors in somatostatin-expressing cells. Our results suggest that somatostatin-expressing cells are resistant to the rate-depressive effects of opioids and that other cells contribute to the effects of fentanyl on breathing. Somatostatin-expressing cells may constitute a cell population that can be targeted to stimulate breathing when it fails with opioids.

## Introduction

Opioid drugs are widely used as pain medications because of their potent analgesic effects. Opioid drugs act on μ-opioid receptors (MORs) in spinal and brainstem regions involved in pain, but also on regions regulating breathing (Stein, 2016; Montandon, 2022). Through their actions on MORs in respiratory circuits, opioid drugs can cause respiratory depression, characterized by slow and shallow breathing, with severe cases involving inhibition of the hypoxic and hypercapnic ventilatory responses and even death with overdose (Dahan et al., 2001; Ramirez et al., 2021). To identify new therapeutic strategies aimed at reducing the side effects of opioid drugs, a better understanding of the mechanisms and respiratory circuits underlying opioid-induced respiratory depression is needed. Many brainstem regions contribute to the respiratory side effects of opioids (Palkovic et al., 2020; Ramirez et al., 2021) including the preBötzinger Complex (preBötC; Montandon et al., 2011; Bachmutsky et al., 2020; Varga et al., 2020a), an inspiratory rhythm-generating site (Smith et al., 1991), the Kölliker–Fuse (KF) nucleus/parabrachial nucleus (PBN; Prkic et al., 2012; Levitt et al., 2015; Miller et al., 2017; Saunders and Levitt, 2020), and the rostral ventromedial medulla (Phillips et al., 2012). The caudal medullary raphe nuclei, including the raphe obscurus (ROb) and raphe pallidus (RPa; Zhang et al., 2007; Phillips et al., 2012; Palkovic et al., 2022) and the nucleus tractus solitarius (NTS; Zhang et al., 2011; Zhuang et al., 2017) may also mediate the severity of opioid-induced respiratory depression because of changes in chemosensory respiratory responses. As well, the nucleus ambiguus (NA) may contribute to changes in the motor control of upper airway muscles (Bieger and Hopkins, 1987; Pascual-Font et al., 2011) associated with opioid drugs (Hassen et al., 1984). Although various brain regions are modulated by opioid drugs and mediate respiratory depression, the identity of the cells mediating these effects is not known.

Brainstem respiratory circuits are identified by various neurotransmitters and neuromodulators. For instance, preBötC neurons express the G-protein-coupled receptor neurokinin-1 receptor and the neuropeptide somatostatin (Stornetta et al., 2003). Although somatostatin is expressed throughout the central and peripheral nervous systems, it acts as a neuromodulator in many brainstem regions controlling breathing (Llona and Eugenín, 2005; Tan et al., 2008; Montandon et al., 2016). In the brainstem, somatostatin is expressed in the preBötC (Stornetta et al., 2003; de Sousa Abreu et al., 2022), KF (De León et al., 1992; Bou Farah et al., 2016), NTS (Helke, 1984; Johansson et al., 1984; Kalia et al., 1984), and NA cells (Johansson et al., 1984), with little expression seen in the raphe nuclei (Johansson et al., 1984). Somatostatin-expressing cells within the preBötC are primarily glutamatergic and involved in modulating the respiratory pattern (Cui et al., 2016; de Sousa Abreu et al., 2022), while their inhibition leads to apnea (Tan et al., 2008), suggesting a critical role of these cells in breathing. Moreover, preBötC somatostatin-expressing cells project to the KF nucleus (Yang and Feldman, 2018), both of which are involved in opioid-induced respiratory depression (Bachmutsky et al., 2020; Varga et al., 2020a). Together these findings suggest that somatostatin-expressing cells are critical for normal respiration and are found within brainstem sites involved in the control of breathing and opioid-induced respiratory depression. We hypothesized that somatostatin and MORs are coexpressed in respiratory circuits and that somatostatin-expressing cells contribute to the regulation of opioid-induced respiratory depression.

Using *in situ* hybridization, we first quantified the expression of *Sst* (gene encoding somatostatin) and *Oprm1* (gene encoding MORs) mRNAs in brainstem regions involved in the control of breathing and respiratory depression. Interestingly, *Oprm1* mRNA expression was observed in the majority (>50%) of *Sst* mRNA-expressing cells in the preBötC, NTS, NA, and KF regions. Next, we determined the role of MORs in the regulation of respiratory depression by opioid drugs by assessing the respiratory responses to fentanyl in wild-type and full MOR knock-out mice (*Oprm1*^−/−^; Matthes et al., 1996). We then generated conditional transgenic knock-out mice that lacked functional MORs only in *Sst*-expressing cells (*Sst-Oprm1*^−/−^). Locomotor responses to fentanyl were also quantified since opioid drugs, such as highly potent fentanyl, have been shown to increase movement in mice, an effect that can impact respiration (Murphy et al., 2001; Contarino et al., 2002; Smith et al., 2009; Varshneya et al., 2019). We found that fentanyl induced a significant respiratory rate depression in wild-type mice, the severity of which was correlated with locomotor hyperactivity, meaning that respiratory rate decreased despite increases in locomotor movements with fentanyl. These responses were absent in *Oprm1*^−/−^ mice, suggesting that MORs fully mediate the effects of fentanyl on respiratory rate and locomotor activity. Surprisingly, mice that lacked functional MORs in *Sst*-expressing cells still presented a significant decline in respiratory rate following systemic fentanyl administration. Our results suggest that, despite coexpression of *Sst* and *Oprm1* mRNAs and the role of *Sst*-expressing cells in the regulation of breathing, these cells are not involved in respiratory rate depression by opioid drugs. Rather, our results suggest that *Sst*-expressing cells involved in breathing may be spared from the effects of opioid drugs.

## Materials and Methods

### Animals and drugs

Mice used in the study were obtained from The Jackson Laboratory and included C57BL/6 (stock #000664), *Oprm1*^−/−^ (stock #007559; Matthes et al., 1996; Martin et al., 2003), *Sst*-Cre (stock #013044; Taniguchi et al., 2011), and *Oprm1*^fl/fl^ (stock #030074). A Cre-*loxP* recombination strategy was used to generate transgenic mice lacking functional MORs in *Sst*-expressing cells by crossing *Sst*-Cre and *Oprm1*^fl/fl^ mice to produce *Sst*-*Oprm1*^−/−^ mice. Custom primers were designed and used to confirm knockout of exons 2 and 3 in the *Oprm1* gene of *Sst-Oprm1*^−/−^ mice using touchdown PCR (Korbie and Mattick, 2008). The *Oprm1* gene in *Oprm1*^fl/fl^ mice was floxed in regions upstream and downstream of exons 2 and 3. Primers were designed to target regions flanking the floxed exon segment. Without the removal of exons 2 and 3 by Cre, the amplicon would be too large (∼3000 bp) to amplify using Taq DNA polymerase when combined with a short synthesis time and therefore no band would be produced (Arezi et al., 2003). After the removal of exons 2 and 3 by Cre, the amplicon would be shorter (∼470 bp), allowing for synthesis and amplification to occur. Different types of tissues were sampled in *Sst*-*Oprm1*^−/−^ mice: ear notch skin tissue as a control and medullary brain tissue, which expresses *Sst* and *Oprm1* (Mansour et al., 1994; Stornetta et al., 2003). Only tissues that coexpress *Sst* (and therefore Cre) and *Oprm1* would have a band, indicating the removal of *Oprm1* exons 2 and 3. Genotyping of *Sst-Oprm1*^−/−^ mice confirmed the presence of *Cre* and *loxP* in ear skin and medullary brain tissue, and the deletion of *Oprm1* exons 2 and 3 in medullary brain tissue, but not in ear skin tissue. Experimental animals were male, 3–4.5 months old, and weighed 20–40 g. Mice were housed with free access to food and water under a 12 h light/dark cycle (lights on at 8:00 A.M.). Fentanyl citrate (50 μg/ml) was purchased from Sandoz.

### *In situ* hybridization

*In situ* hybridization was performed in *Sst*-Cre mice to determine expression of *Sst* and *Oprm1* mRNAs in brainstem regions involved in respiration. Mice were perfused with PBS followed by formalin, and the brain was harvested. Brains were soaked in 20% sucrose in PBS for 24 h followed by 30% sucrose in PBS for 24 h. Fixed brains were frozen using Tissue-Tek O.C.T. Compound (Sakura) and dry ice and were stored at −80°C. Coronal sections were cut at 25 μm thickness using a cryostat (model CM3050S, Leica Biosystems). The manufacturer protocol was used to perform *in situ* hybridization using the RNAscope Multiplex Fluorescent Reagent version 2 Assay (ACD; Wang et al., 2012), and sections were counterstained with DAPI. Target probes used were Mm-*Sst* (catalog #404631-C3, ACD) targeting *Sst* gene mRNA and Mm-*Oprm1* (catalog #315841, ACD) targeting *Oprm1* gene mRNA. Tissue sections 100 μm apart were scanned using the Axio Scan.Z1 slide scanner (Zeiss).

### mRNA quantification

To quantify mRNA expression, two to three sections containing each region of interest were exported from Zen (Zeiss) to Adobe Illustrator (Creative Suite 5, Adobe). Regions where mRNA was quantified include the preBötC, NTS, ROb and RPa nuclei, NA, and KF nucleus. To identify these areas in tissue sections, *The Mouse Brain in Stereotaxic Coordinates* (Franklin and Paxinos, 2008) was consulted, and anatomic markers included the NTS, the NA, the facial nucleus, the hypoglossal nucleus, the cerebellum, and the external cuneate nucleus. Regions of interest were drawn, and images were exported to Fiji (ImageJ) for counting. We used the manufacturer *Guide for RNAscope Data Analysis* (ACD) to count and distinguish cell mRNA expression from background expression. Cells were considered to express mRNA if four or more dots (one dot represents one mRNA molecule) and/or one or more clusters of dots overlapped with or were adjacent to a DAPI-stained cell nucleus. Coexpression of *Sst* and *Oprm1* mRNA was considered if four or more dots and/or one or more clusters of dots for both *Sst* and *Oprm1* mRNA overlapped with or were adjacent to a DAPI-stained cell nucleus. Counts were obtained for total DAPI, *Oprm1*, *Sst*, and *Oprm1 *+* Sst* cells, and expression was presented as percentages of total DAPI, total *Oprm1*, and total *Sst* cells.

### Whole-body plethysmography

Flow-through whole-body plethysmography was used to measure respiratory activity in freely moving mice (Buxco Bias Flow Manual, DSI), according to previous studies (Montandon et al., 2006; Bachmutsky et al., 2020). Eight C57BL/6, seven *Oprm1*^−/−^, nine *Sst*-Cre, and eight *Sst*-*Oprm1*^−/−^ mice were used. Plethysmography chambers were ventilated with a constant airflow of 0.9 L/min at room temperature and measured 21.5 cm in diameter, allowing enough room for mice to move freely during recordings. Pressure changes inside the chamber were recorded with a pressure transducer, amplified (model PS100W-2, EMKA Technologies), and digitized using PowerLab 4/26 in LabChart Version 8 (ADInstruments). Data were extracted from LabChart Version 8 (ADInstruments) and exported to Microsoft Excel for analysis. To approximate tidal volume, we calculated the area under the curve during inspiration using pressure traces. Since accurate approximation of tidal volume requires measurements of body temperature, chamber humidity, and temperature (Mortola and Frappell, 1998), which is challenging and often inaccurate in small rodents (Enhorning et al., 1998), we normalized tidal volume according to baseline values, expressed it as percentage of baseline, and labeled it relative tidal volume. We thereby assumed that temperatures and humidity did not change substantially during the experiment. In addition, considering the behavioral changes observed following saline or fentanyl injection, relative tidal volume measured with this approach includes behavioral artifacts such as sniffing and movements that may affect the reliability of tidal volume measurements. We measured respiratory rate as the number of breaths per minute. Relative minute ventilation was calculated as the product of relative tidal volume and respiratory rate.

Mice were acclimatized to the plethysmography chamber for 3 d before experiments between 10:00 A.M. and 1:00 P.M. Experiments took place over 2 d at the same time of day as the acclimatization period. During the 2 experimental days, mice were placed in the plethysmography chamber, and baseline measures were recorded. At 12:00 P.M., mice received an intraperitoneal injection of either saline or fentanyl citrate (0.3 mg/kg; Fujii et al., 2019). The volume injected to achieve a dose of 0.3 mg/kg fentanyl was calculated to be 120 μl/20 g mouse. The injected volumes of saline and fentanyl were calculated on each respective experimental day based on the weight of the mouse obtained at 10:00 A.M. and ranged between 120 and 240 μl, depending on the weight of the mouse. Treatments were randomized between experimental days. Data for 1 h preinjection and 1 h postinjection were divided into 1 min average time bins and used to assess respiratory parameters over time. Respiratory variables were averaged for the 1 h period leading up to injection on the first experimental day, to determine baseline measures. Averages of respiratory rate, relative tidal volume, and relative minute ventilation were obtained for minutes 5–10 and 50–55 postinjection to assess representative 5 min windows during “early” and “late” phases, respectively, of the postinjection response. Representative 5 s traces of respiratory recordings were extracted beginning at minute 7 postinjection, during the peak effect of fentanyl.

### Locomotor activity

Whole-body plethysmography chambers with transparent bottom platforms were mounted on a box with a 1080p high-definition camera placed facing upward at the bottom of the structure. This camera allowed for simultaneous video recording of mouse movements during respiratory recordings. Mouse movements were tracked during both experimental days for the entire 3 h duration. Videos were recorded using Pinnacle Studio 24 MultiCam Capture software (Corel), resized, and exported to EthoVision XT Version 14 (Noldus) for movement analysis. Mouse velocity was calculated for minutes 5–10 and 50–55 postinjection in Microsoft Excel, and video data were aligned with respiratory recordings using video time stamps.

### Statistics

Statistical analyses were performed using GraphPad Prism 9. Data in all figures and text are represented as the mean ± SEM with individual data points displayed. Data were tested for normality using the Shapiro–Wilk test. Normally distributed data were analyzed using an unpaired *t* test with Welch’s correction, two-way repeated-measures ANOVA, or a mixed-effects model, to account for missing values. The repeated measure in this study was drug treatment (saline or fentanyl). A Sidak multiple-comparisons *post hoc* test was used following the two-way repeated-measures ANOVA or mixed-effects analysis. Non-normally distributed data were analyzed using the Mann–Whitney test or the Wilcoxon matched-pairs signed-rank test with the Holm–Sidak multiple-comparisons *post hoc* test. Pearson’s correlation coefficient was used to determine the association between respiratory rate and velocity. All tests were two tailed with a significance level of α = 0.05.

## Results

### Expression of *Sst* and *Oprm1* mRNAs

**Figure 1.**
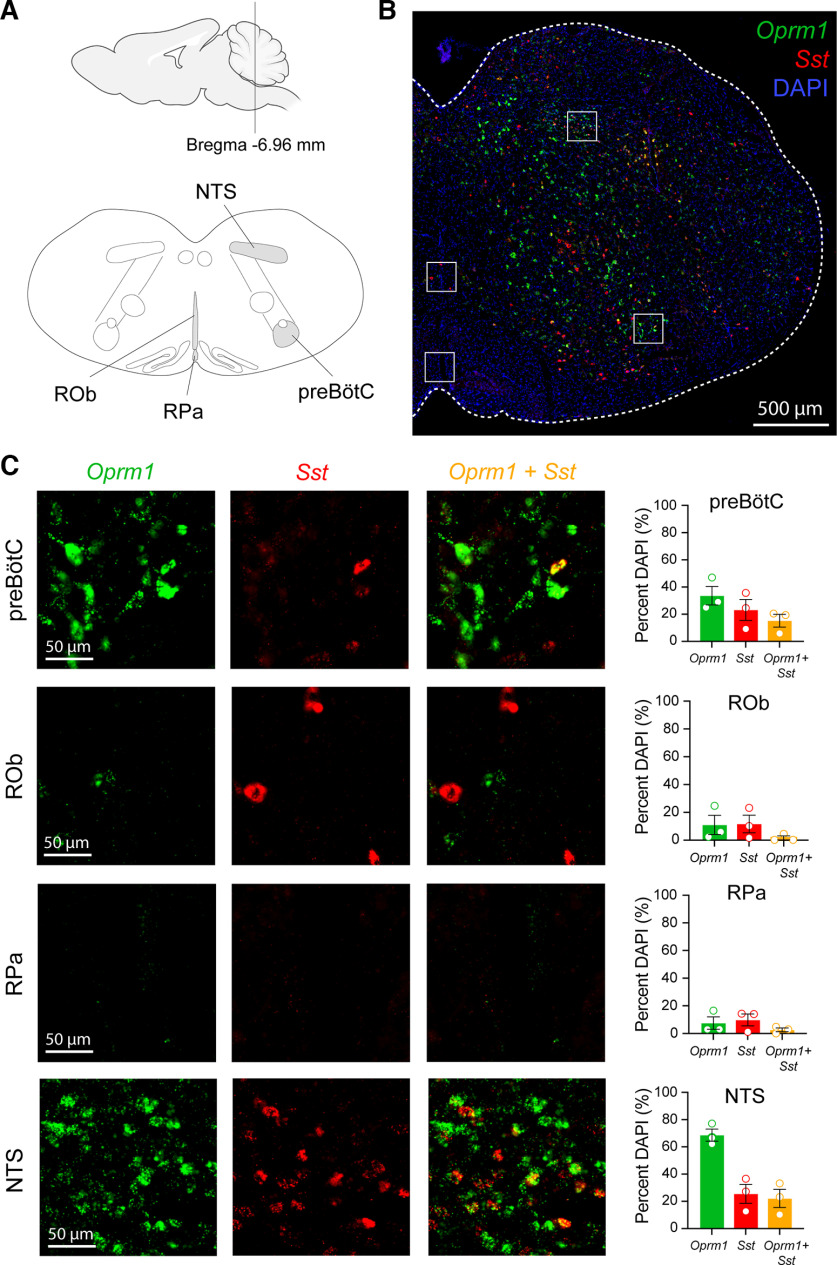
Expression of *Sst* and *Oprm1* mRNAs in respiratory medullary circuits. ***A***, Diagrams of medullary sections located 6.96 mm caudal to bregma. ***B***, Whole brainstem section of a control (*Sst*-Cre) mouse showing DAPI (blue), *Oprm1* mRNA (green), and *Sst* mRNA (red) expression. White squares indicate the regions of interest shown in ***C***. Scale bar, 500 μm. ***C***, *Sst* and *Oprm1* mRNA expression in the preBötC, the ROb, the RPa, and the NTS. Scale bar, 50 μm. *Sst* and *Oprm1* mRNA were quantified and expressed as a percentage of total DAPI cells (right panels). Bars represent the mean ± SEM.

**Figure 2.**
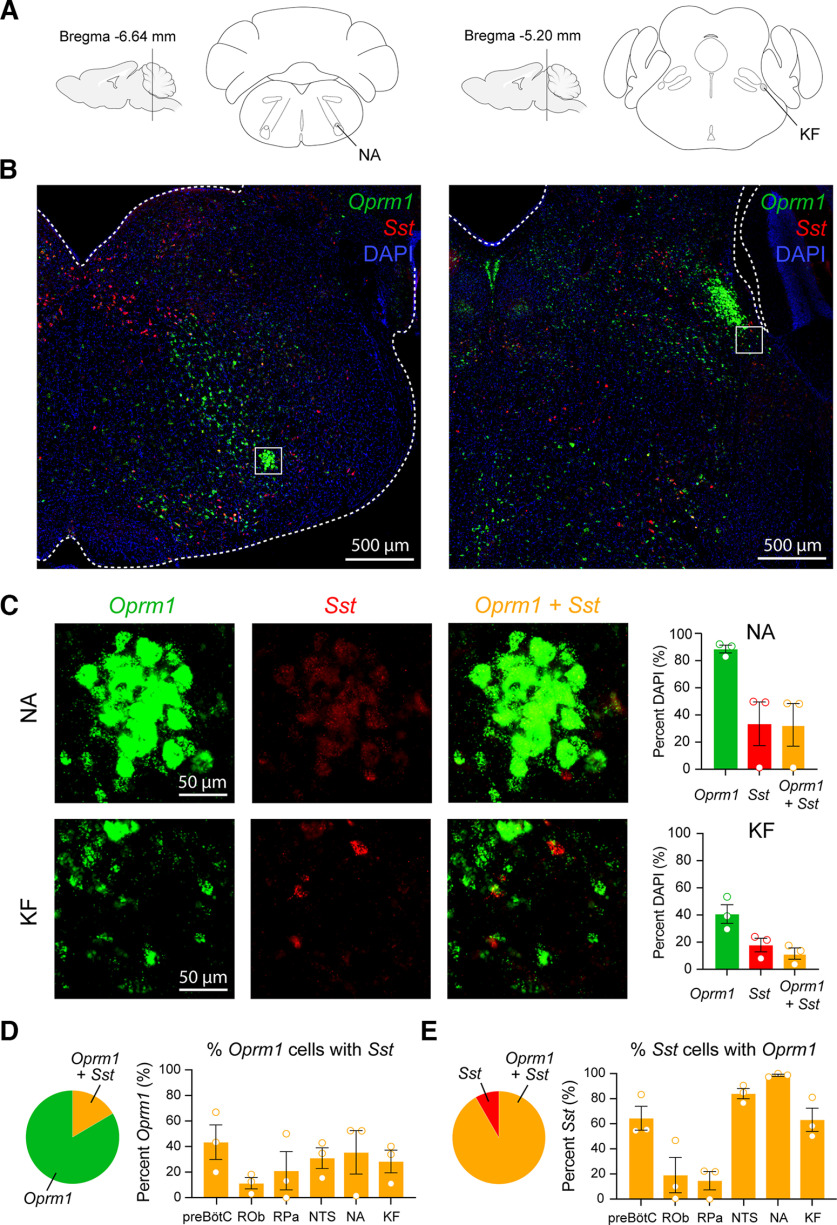
Expression of *Sst* and *Oprm1* mRNAs in respiratory medullary and pontine circuits. ***A***, Diagrams of brainstem sections located 6.64 and 5.20 mm caudal to bregma. ***B***, DAPI (blue), *Oprm1* mRNA (green), and *Sst* mRNA (red) expression in whole brainstem sections of control (*Sst*-Cre) mice. White squares indicate regions of interest. Scale bar, 500 μm. ***C***, *Sst* and *Oprm1* expression in the NA and the KF nucleus. Scale bar, 50 μm. *Sst* and *Oprm1* mRNA quantified and expressed as a percentage of total DAPI cells (right panels). ***D***, Relative coexpression of *Oprm1* and *Sst* as a percentage of *Oprm1* cells in the preBötC, the ROb, the RPa, the NTS, the NA, and the KF nucleus. ***E***, Relative coexpression of *Oprm1* and *Sst* as a percentage of *Sst* cells. Bars represent the mean ± SEM.

To determine the expression of *Sst* and *Oprm1* mRNAs, we performed *in situ* hybridization in medullary and pontine regions of control (*Sst*-Cre) mice (*n* = 3). We first analyzed sections located 6.8–7.1 mm caudal to bregma containing the preBötC, ROb, RPa, and the NTS (Fig. 1*A*,*B*). In the preBötC, 33.6 ± 6.8% of DAPI-stained cells expressed *Oprm1*, 23.1 ± 7.7% expressed *Sst*, and 15.2 ± 4.7% coexpressed *Oprm1* and *Sst* (Fig. 1*C*). In the ROb, *Oprm1* mRNA was expressed in 11.0 ± 6.9% of DAPI-stained cells, and *Sst* was expressed in 11.7 ± 6.3% of DAPI-stained cells; 1.7 ± 1.4% of DAPI-stained cells in the ROb coexpressed both *Oprm1* and *Sst*. In the RPa, 7.5 ± 4.5% of DAPI-stained cells expressed *Oprm1*, 9.8 ± 4.3% expressed *Sst*, and 2.6 ± 1.4% coexpressed *Sst* and *Oprm1*. In the NTS, *Oprm1* mRNA was expressed in 68.6 ± 4.5% of DAPI-stained cells, whereas *Sst* mRNA was expressed in 25.5 ± 7.0% of DAPI-stained cells. Coexpression of both *Oprm1* and *Sst* mRNAs in the NTS was found in 22.2 ± 6.6% of DAPI-stained cells. We then looked at the NA, located 6.6–6.7 mm caudal to bregma (Fig. 2*A–C*). *Oprm1* was expressed in 88.5 ± 2.8% of DAPI-stained cells, *Sst* was expressed in 33.5 ± 16.1% of DAPI-stained cells, and both *Oprm1* and *Sst* were found in 32.7 ± 15.7% of DAPI-stained cells. Last, we looked at mRNA expression in the KF nucleus, 4.8–5.3 mm caudal to bregma (Fig. 2*A–C*): 40.7 ± 6.9% of DAPI-stained cells expressed *Oprm1*, 17.9 ± 5.0% expressed *Sst*, and 11.7 ± 4.1% expressed both *Oprm1* and *Sst.* In summary, relatively high expression of *Oprm1* mRNA was found in the NTS and the NA, with moderate expression in the preBötC and the KF nucleus, and low expression in the raphe nuclei. *Sst* mRNA was moderately expressed in the preBötC, the NTS, the NA, and the KF nucleus, with relatively low expression in the caudal raphe nuclei.

To better assess the relationship between *Oprm1* and *Sst* expression, we looked at the relative proportion of *Oprm1 *+* Sst* cells among *Oprm1*-expressing cells (Fig. 2*D*). In other words, how many *Oprm1* cells expressed *Sst* mRNA? A total of 43.4 ± 13.5% of *Oprm1*-expressing cells coexpressed *Sst* in the preBötC, 11.2 ± 4.4% in the ROb, 21.1 ± 15.0% in the RPa, 31.0 ± 8.1% in the NTS, 35.4 ± 17.0% in the NA, and 28.3 ± 8.9% in the KF nucleus. We also looked at the relative proportion of *Oprm1 *+* Sst* mRNAs among *Sst*-expressing cells (Fig. 2*E*): 64.4 ± 9.5% of *Sst*-expressing cells coexpressed *Oprm1* in the preBötC, 19.1 ± 14.1% in the ROb, 14.7 ± 7.3% in the RPa, 84.0 ± 4.1% in the NTS, 98.7 ± 0.8% in the NA, and 63.2 ± 9.3% in the KF nucleus. In conclusion, relatively high expression of *Oprm1*-expressing cells was found in the NTS and NA, moderate expression in the preBötC and the KF nucleus, and low expression in the ROb and RPa nuclei. While *Sst* expression was low to moderate in all regions, a majority (>50%) of *Sst*-expressing cells in the preBötC, the NTS, the NA, and the KF nucleus coexpressed *Oprm1*. Interestingly, >20% of *Oprm1*-expressing cells in the preBötC, the NTS, and the KF nucleus coexpressed *Sst*. Considering the role of the preBötC and the KF nucleus in mediating opioid-induced respiratory depression (Montandon et al., 2011; Varga et al., 2020a), our findings suggest that the *Sst* cell populations in these regions may contribute to respiratory depression by opioid drugs.

### Deletion of MORs and respiratory depression by the opioid fentanyl

**Figure 3.**
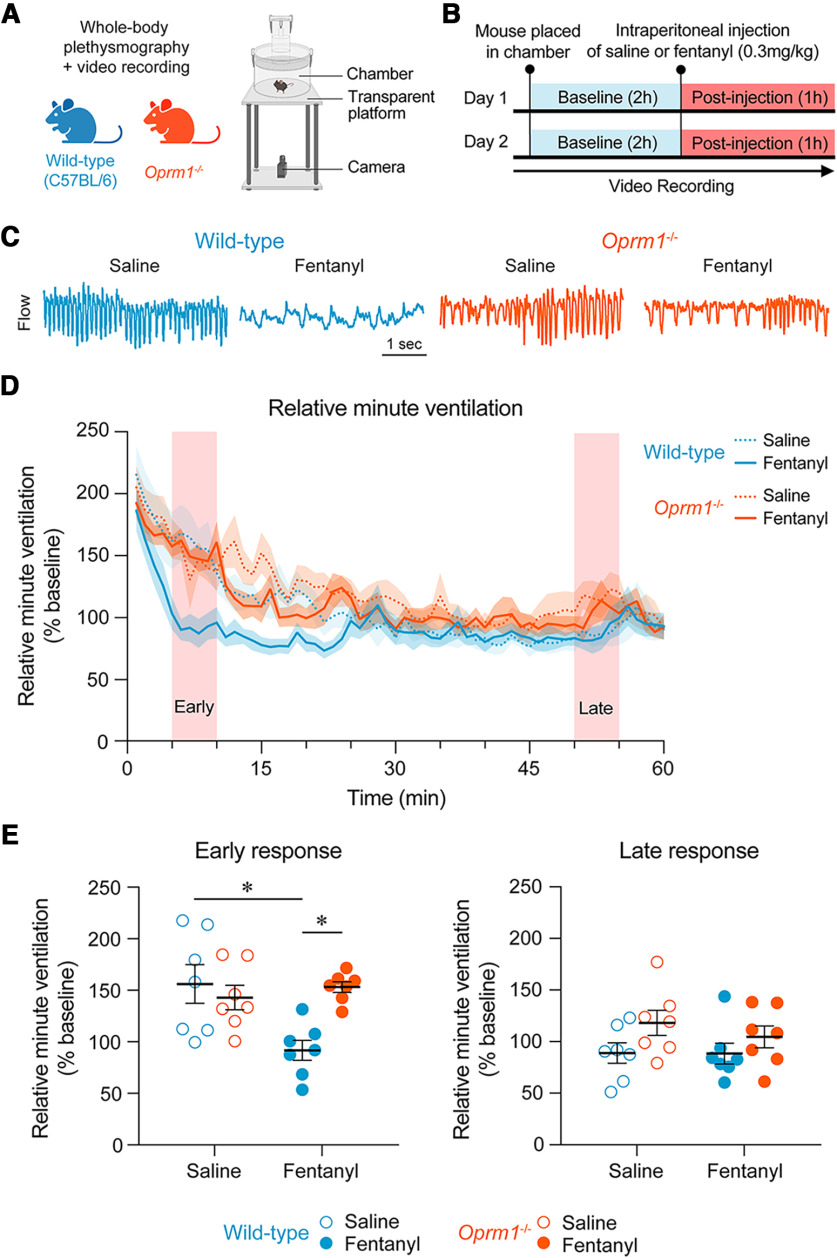
Opioid-induced respiratory depression in wild-type and *Oprm1*^−/−^ mice. ***A***, Using a combination of whole-body plethysmography and video recordings, respiratory and locomotor activities were recorded in wild-type and *Oprm1*^−/−^ mice. ***B***, On day 1, mice were placed in the chamber and respiratory rate was recorded for 2 h. An intraperitoneal injection of either saline or fentanyl (0.3 mg/kg) was administered after 2 h of baseline activity. Responses were then recorded for 1 h. On day 2, mice were placed in the chamber and recorded for 2 h followed by an intraperitoneal injection of either fentanyl (0.3 mg/kg) or saline, and respiratory activity was recorded for 1 h. ***C***, Representative traces of respiratory responses to saline and fentanyl in wild-type and *Oprm1*^−/−^ mice. ***D***, Relative minute ventilation following injection of saline and fentanyl in wild-type and *Oprm1*^−/−^ mice. ***E***, Relative minute ventilation averaged over a 5 min period during the early phase and late phase of the responses to saline and fentanyl. Data are represented as the mean ± SEM. *p < 0.05. Panel ***A*** was created with BioRender.com.

To characterize the effects of fentanyl on respiratory activity, we administered a single dose of fentanyl (0.3 mg/kg; Fujii et al., 2019) in wild-type (C57BL/6) and *Oprm1*^−/−^ knock-out mice, and measured breathing using whole-body plethysmography (Fig. 3*A*,*B*). The first 5 min following injection were omitted from all analyses to allow for the drug to take effect and for the mouse to adjust following injection. Respiratory responses to fentanyl and saline were calculated and analyzed for the early and late phases of the response. Systemic injection of fentanyl decreased relative minute ventilation compared with saline in wild-type mice, but not in *Oprm1*^−/−^ mice (Fig. 3*C*,*D*). During the early phase (minutes 5–10 postinjection), there was a significant interaction between treatment (saline or fentanyl) and genotype (*p* = 0.0041, mixed-effects model with Sidak multiple-comparisons test; Fig. 3*E*). Fentanyl induced a significant decrease in relative minute ventilation compared with saline in wild-type mice (*p* = 0.0022) but not in *Oprm1*^−/−^ mice (*p* = 0.7535). During this early phase, relative minute ventilation was significantly lower in wild-type mice compared with *Oprm1*^−/−^ mice following fentanyl injection (*p* = 0.0026). During the late phase (minutes 50–55 postinjection), there were no significant differences between relative minute ventilation in response to fentanyl compared with saline in wild-type mice (*p* > 0.9999, Wilcoxon matched-pairs signed-rank test with Holm–Sidak multiple comparisons) or *Oprm1*^−/−^ mice (*p* = 0.9961).

**Figure 4.**
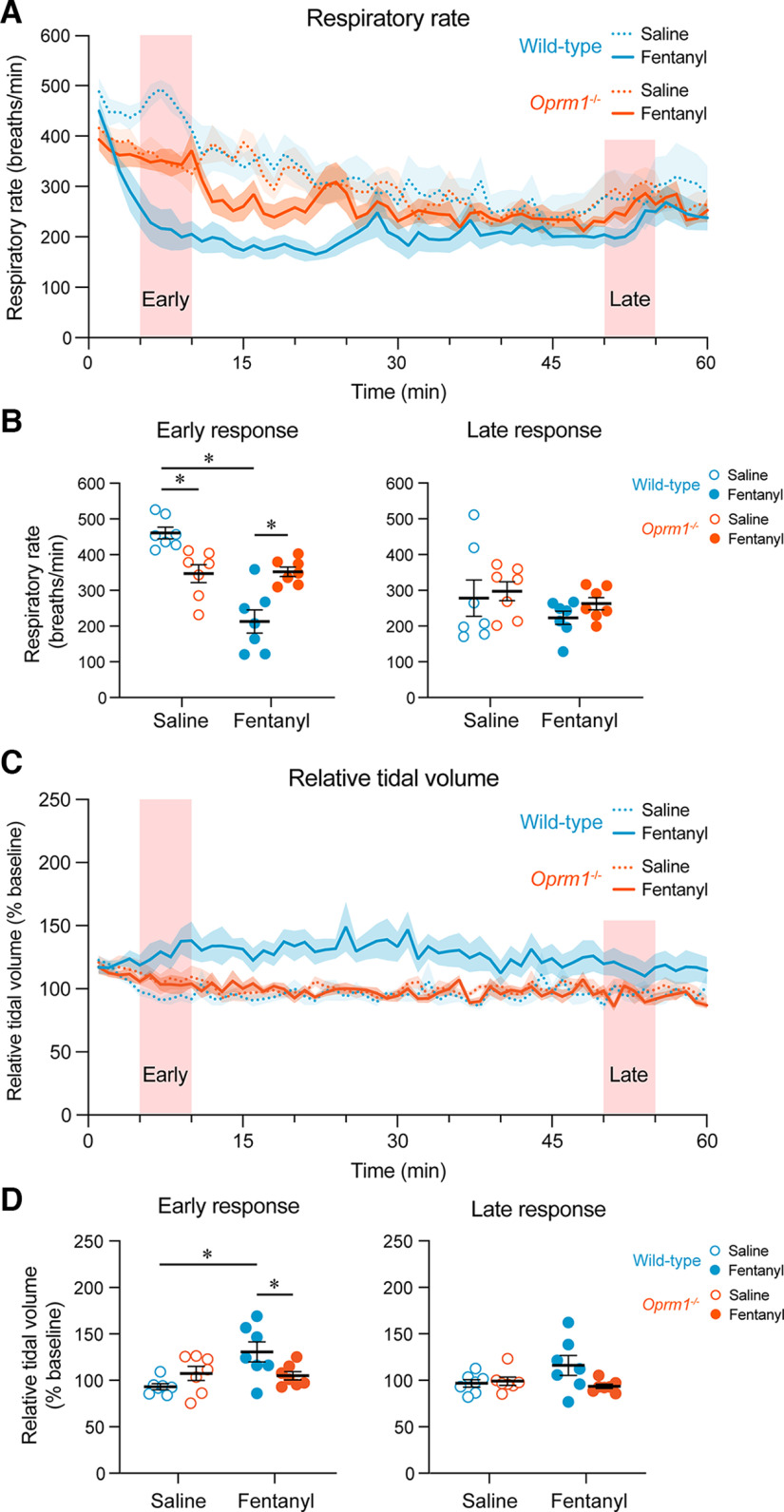
Respiratory rate and relative tidal volume in response to fentanyl in wild-type and *Oprm1*^−/−^ mice. ***A***, Respiratory rate following the injection of saline or fentanyl in wild-type and *Oprm1*^−/−^ mice. ***B***, Respiratory rate averaged over a 5 min period during the early and late phase of the responses to saline and fentanyl. ***C***, Relative tidal volume following the injection of saline or fentanyl. ***D***, Relative tidal volume averaged over a 5 min period during the early phase and late phase of the responses to saline and fentanyl.

Systemic injection of fentanyl decreased respiratory rate relative to saline injection in wild-type mice but not in *Oprm1*^−/−^ mice (Fig. 4*A*). During the early phase, there was a significant interaction between treatment (saline or fentanyl) and genotype (*p* < 0.0001, mixed-effects model with Sidak multiple comparisons). Fentanyl induced a significant decline in respiratory rate relative to saline in wild-type mice (*p* < 0.0001) but not in *Oprm1*^−/−^ mice (*p* = 0.9845; Fig. 4*B*). During this early phase, respiratory rate was significantly greater in wild-type mice compared with *Oprm1*^−/−^ mice following saline injection (*p* = 0.0038), but was significantly greater in *Oprm1*^−/−^ mice compared with wild-type mice following fentanyl injection (*p* = 0.0005). During the late phase, there were no significant differences between respiratory rate in response to fentanyl compared with saline in wild-type mice (*p* > 0.9999, Wilcoxon matched-pairs signed-rank test with Holm–Sidak multiple comparisons) or *Oprm1*^−/−^ mice (*p* = 0.5056). No significant differences were found between baseline respiratory rates of wild-type and *Oprm1*^−/−^ mice (*p* = 0.7789, Mann–Whitney test).

Interestingly, relative tidal volume increased in response to fentanyl when compared with saline in wild-type mice, but not in *Oprm1*^−/−^ mice (Fig. 4*C*). During the early phase, there was a significant interaction between treatment (saline or fentanyl) and genotype (*p* = 0.0059, mixed-effects model with Sidak multiple comparisons; Fig. 4*D*), where fentanyl significantly increased relative tidal volume in wild-type mice (*p* = 0.0019) but not in *Oprm1*^−/−^ mice (*p* = 0.9514). Relative tidal volume was also significantly greater in wild-type mice compared with *Oprm1*^−/−^ mice in response to fentanyl (*p* = 0.0339). No significant interaction occurred between treatment (saline or fentanyl) and genotype (*p* = 0.0573, mixed-effects model) during the late phase in wild-type or *Oprm1*^−/−^ mice, and no significant effects of treatment (*p* = 0.2740) or genotype (*p* = 0.1160) were found. Overall, our findings demonstrate that MORs regulate respiratory depression by fentanyl in freely behaving mice, mainly because of reductions in respiratory rate induced by fentanyl.

### Deletion of MORs and locomotor activity

**Figure 5.**
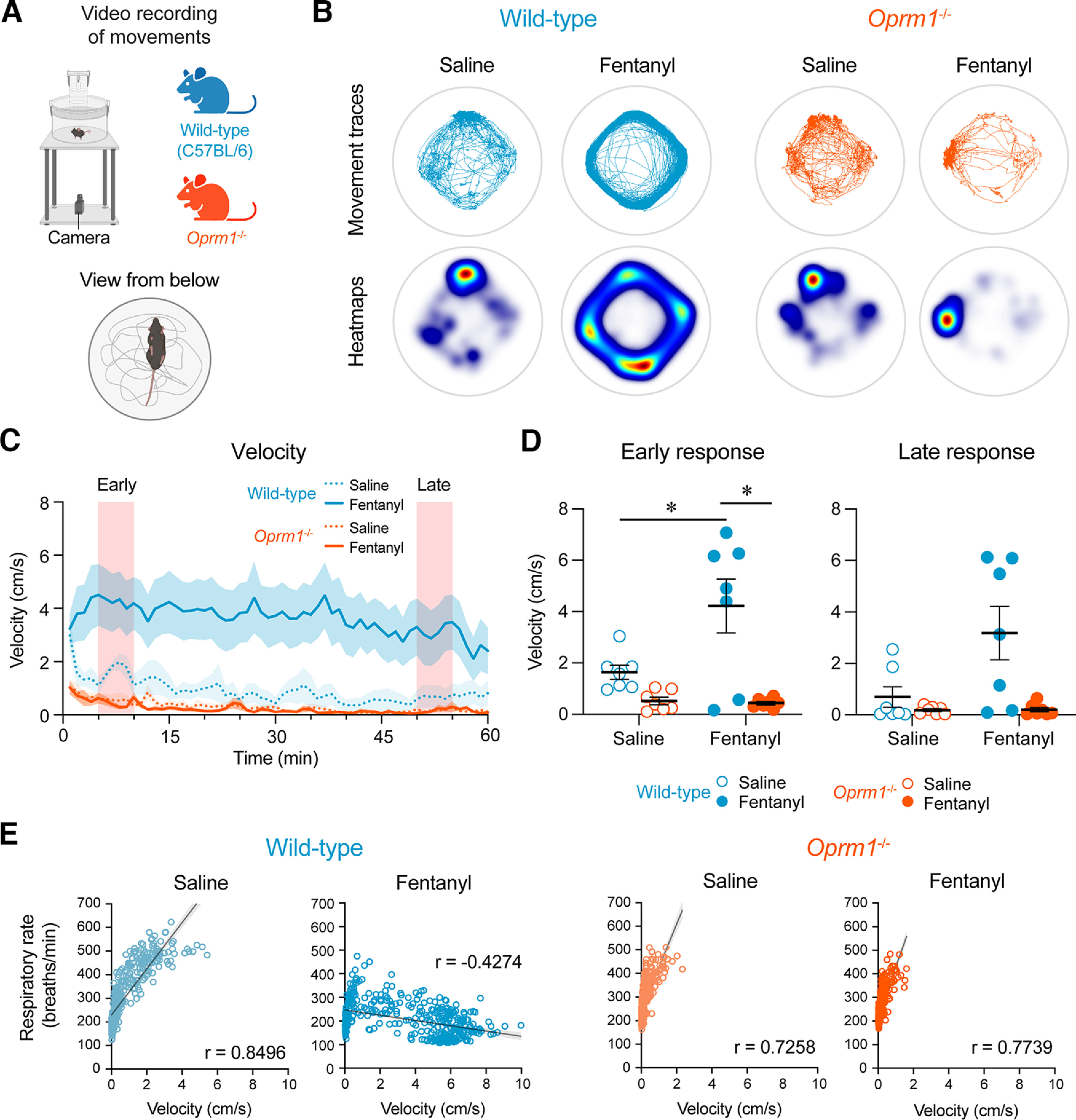
Opioid-induced locomotor hyperactivity in wild-type and *Oprm1*^−/−^ mice. ***A***, Videos were recorded from the bottom of the plethysmography chamber to assess mouse movements. ***B***, Representative tracings and heat maps of mouse movements during the 1 h postinjection period. ***C***, Average velocity for 1 h postinjection of saline and fentanyl in wild-type and *Oprm1*^−/−^ mice. ***D***, Velocity during early and late responses to injections. ***E***, Relationships between velocity and respiratory rate in wild-type and *Oprm1*^−/−^ mice following saline and fentanyl injections. Data are represented as the mean ± SEM. *p < 0.05. Panel ***A*** was created with BioRender.com.

Opioid drugs such as fentanyl produce profound effects on locomotor activity in mice (Murphy et al., 2001; Contarino et al., 2002; Smith et al., 2009; Varshneya et al., 2019). One limitation associated with respiratory recordings in freely behaving mice is that the plethysmography recording system also captures movements and cannot distinguish between various behavioral states, which may directly impact breathing (Montandon and Horner, 2019). To better understand the effects of fentanyl on respiration, we quantified its effects on locomotion and examined the relationship between locomotor activity and respiration in wild-type and *Oprm1*^−/−^ mice (Fig. 5*A*). Baseline average velocities (movement of the mouse per second) showed no significant differences between wild-type and *Oprm1*^−/−^ mice (*p* = 0.4634, Mann–Whitney test). Representative movement traces of wild-type and *Oprm1*^−/−^ mice following injection of either saline or fentanyl are shown in Figure 5*B*. Velocity increased in wild-type mice following systemic injection of fentanyl when compared with saline, but no change in velocity was observed in *Oprm1*^−/−^ mice (Fig. 5*C*). The postinjection response was divided into early and late phases (Fig. 5*D*). During the early phase (minutes 5–10 postinjection), a significant interaction occurred between treatment (saline or fentanyl) and genotype (*p* = 0.0192, mixed-effects model with Sidak multiple comparisons). Fentanyl significantly increased velocity compared with saline in wild-type mice (*p* = 0.0069), but not in *Oprm1*^−/−^ mice (*p* = 0.9898). Velocity was also significantly increased following fentanyl injection in wild-type mice compared with *Oprm1*^−/−^ mice (*p* = 0.0001). During the late phase (minutes 50–55 postinjection), no significant differences were found for velocity in response to fentanyl when compared with saline in wild-type or *Oprm1*^−/−^ mice (*p* = 0.1787 and *p* = 0.9375, respectively, Wilcoxon matched-pairs signed-rank test with Holm–Sidak multiple comparisons). Overall, fentanyl significantly increased locomotion in wild-type mice and had no effect on locomotion in *Oprm1*^−/−^ mice, suggesting that MORs mediate the locomotor response to fentanyl.

Next, we examined the association between velocity and respiratory rate in response to the injection of saline and fentanyl (Fig. 5*E*). Significant positive correlations were found between velocity and respiratory rate in both wild-type mice (*r* = 0.8496, *p* < 0.0001) and *Oprm1*^−/−^ mice (*r* = 0.7258, *p* < 0.0001) in response to saline injection, for which increased velocity was associated with increased respiratory rate. Following fentanyl injection, a significant negative correlation was found between velocity and respiratory rate in wild-type mice (*r* = −0.4274, *p* < 0.0001), where greater velocity was associated with lower respiratory rate. However, a positive correlation was found between respiratory rate and velocity in *Oprm1*^−/−^ mice following fentanyl injection (*r* = 0.7739, *p* < 0.0001). These results show that, despite substantial locomotor activity in mice following fentanyl injection, respiratory rate was depressed, and, interestingly, its severity increased in association with increased movement. By contrast, a positive correlation was found between velocity and respiratory rate following fentanyl injection in *Oprm1*^−/−^ mice, suggesting that the loss of functional MORs abolished the effects of fentanyl.

### Deletion of MORs in *Sst*-expressing cells and respiratory depression by fentanyl

**Figure 6.**
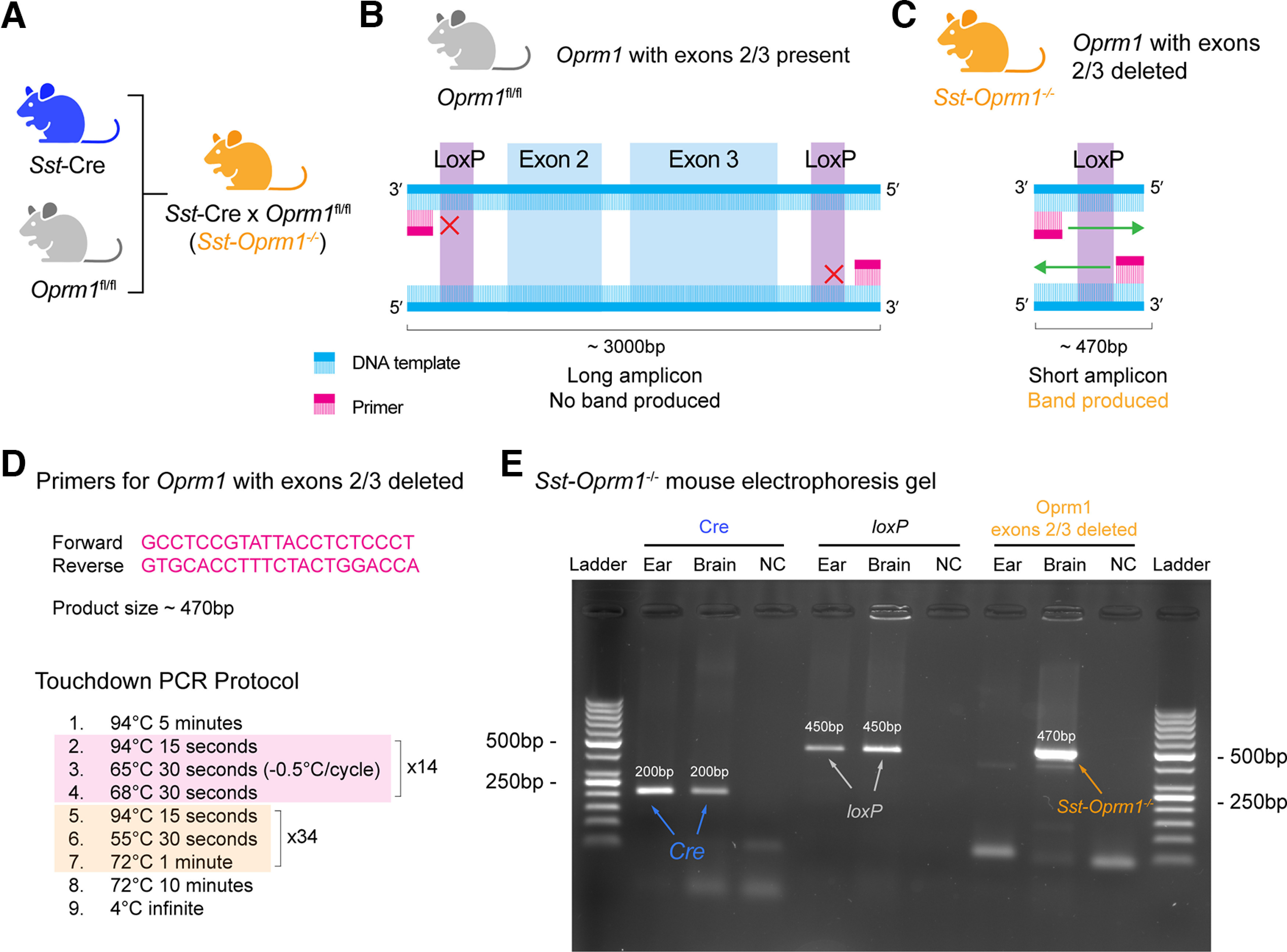
Genotyping of conditional *Sst-Oprm1*^−/−^ mice. ***A***, To produce *Sst-Oprm1*^−/−^ mice, we bred *Oprm1*^fl/fl^ and *Sst*-Cre mice. ***B***, In *Oprm1*^fl/fl^ mice, the sequence of the floxed *Oprm1* exons 2 and 3 was ∼3000 bp in length. ***C***, When exons 2 and 3 of the *Oprm1* gene were removed, the sequence was reduced to ∼470 bp. Taq DNA polymerase can synthesize DNA at a rate of ∼1000 bp/min (Arezi et al., 2003; Korbie and Mattick, 2008). When provided with a short synthesis time (<1 min) during the PCR protocol, the long fragment would be too long to amplify, therefore resulting in no band being produced. ***D***, To determine whether the *Oprm1* gene was deleted specifically in *Sst*-expressing cells, we genotyped tissue with coexpression of *Sst* and *Oprm1*, such as medullary brain tissue, and tissue without coexpression, such as the ear skin in *Sst-Oprm1*^−/−^ mice. Using custom primers, the short amplicon of 470 bp was amplified by Taq DNA polymerase using a short synthesis time. Sequences are provided for the forward and reverse primers designed to target regions upstream and downstream of the floxed exon 2 and 3 fragment of the *Oprm1* gene. The protocol used for touchdown PCR is shown along with associated cycle numbers and durations (Korbie and Mattick, 2008). ***E***, Electrophoresis gel from an *Sst-Oprm1*^−/−^ mouse with tissue samples from an ear notch and the medulla indicates bands with Cre (∼200 bp) and *loxP* (∼450 bp) in both ear and medullary samples. A band for *loxP* was produced in *Sst-Oprm1*^−/−^ mice since the sequence was still present in non-*Sst*-expressing cells, and one *loxP* sequence also remained after the excision of *Oprm1* exons 2 and 3 by Cre. The presence of a mutant band using our custom primers (∼470 bp) indicated that the floxed *Oprm1* exon 2 and 3 fragment was excised in medullary tissue but not in ear notch tissue. Negative controls (NCs) are indicated.

To determine the role of *Sst*-expressing cells in opioid-induced respiratory depression, we produced conditional transgenic knock-out mice that lacked functional MORs in *Sst*-expressing cells only (Fig. 6). To delete exons 2 and 3 of the *Oprm1* gene in *Sst*-expressing cells, we used a Cre-*loxP* recombination strategy and bred *Sst*-Cre and *Oprm1*^fl/fl^ mice to produce *Sst*-*Oprm1*^−/−^ mice. Although breeding strategies with Cre-*loxP* recombination are well established and are commonly used, especially in the context of *Oprm1* knockout (Severino et al., 2020), we designed a genotyping protocol to confirm that the *Oprm1* gene was deleted in *Sst*-expressing cells. Breeding of *Sst*-Cre and *Oprm1*^fl/fl^ mice aimed to remove exons 2 and 3 of the *Oprm1* gene (Fig. 6*A*). In *Oprm1*^fl/fl^ mice, the amplicon of the floxed *Oprm1* exons 2 and 3 was ∼3000 bp in length (Fig. 6*B*). When exons 2 and 3 of the *Oprm1* gene were removed, the amplicon was reduced to ∼470 bp (Fig. 6*C*). Thus, the size of the amplicon should be 470 bp in *Sst*-expressing cells of *Sst*-*Oprm1*^−/−^ mice. Considering that Taq DNA polymerase can synthesize DNA at a rate of ∼1000 bp/min (Arezi et al., 2003; Korbie and Mattick, 2008), we used a short synthesis time (<1 min) during the PCR protocol, so only the short amplicon of 470 bp was amplified (Fig. 6*D*). Electrophoresis gel from tissue samples from an ear notch and the medulla of a *Sst-Oprm1*^−/−^ mouse indicated the presence of Cre (∼200 bp) and *loxP* (∼450 bp) in both ear and medullary samples (Fig. 6*E*). Importantly, a mutant band using our primer (∼470 bp) was found in medullary tissue but not ear notch tissue, suggesting tissue-specific excision based on the coexpression of *Sst* and *Oprm1*. In conclusion, our genotyping approach confirmed that *Sst*-*Oprm1*^−/−^ mice lacked *Oprm1* exons 2 and 3 in *Sst*-expressing cells.

**Figure 7.**
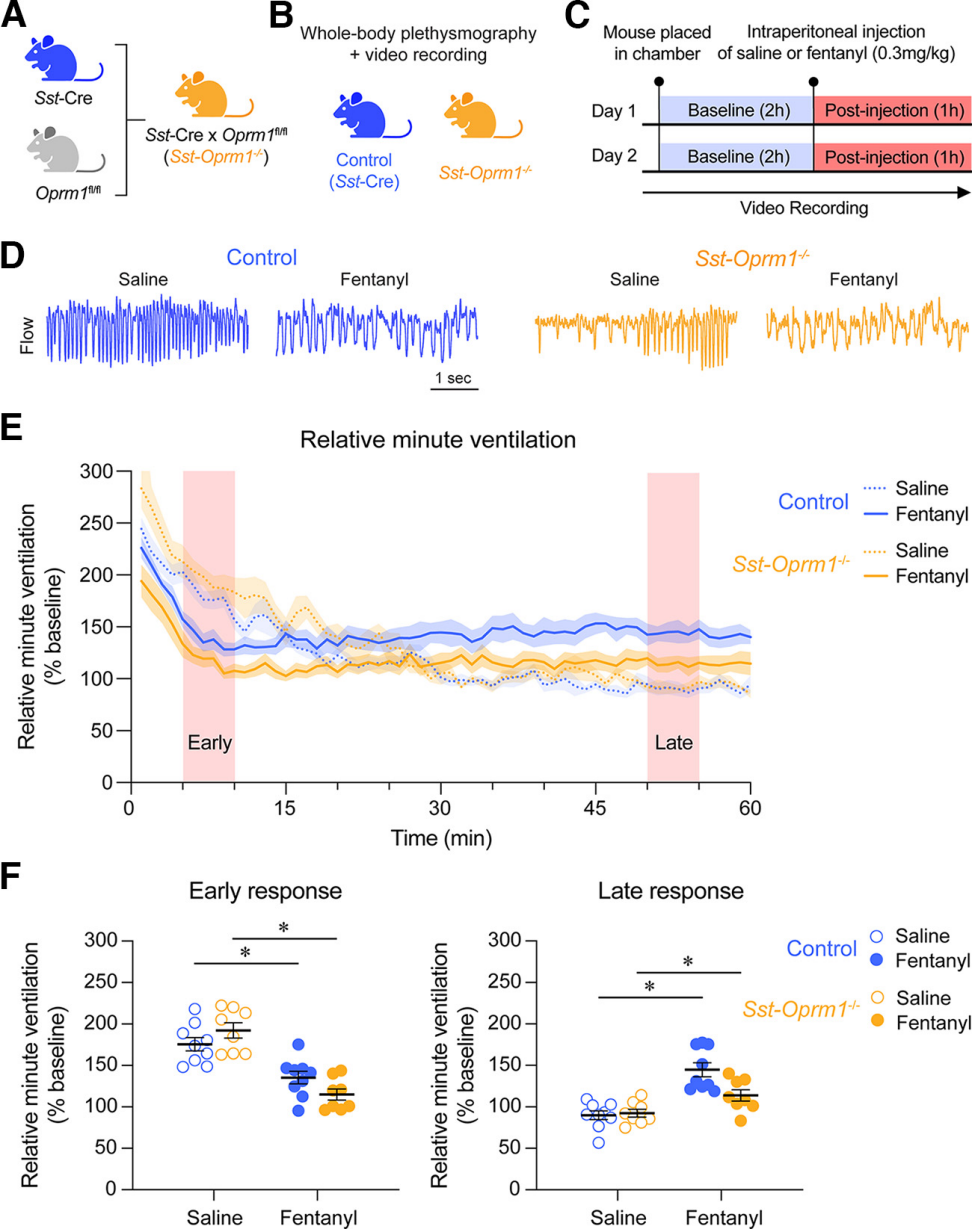
Opioid-induced respiratory depression in control and *Sst-Oprm1*^−/−^ mice. ***A***, Generation of *Sst-Oprm1*^−/−^ mice using a Cre-*loxP* recombination strategy by breeding *Oprm1*^fl/fl^ and *Sst*-Cre mice. ***B***, ***C***, Respiratory parameters were measured using whole-body plethysmography recording in freely behaving control (*Sst*-Cre) and *Sst-Oprm1*^−/−^ mice. ***D***, Representative traces of respiratory responses to saline and fentanyl injections. ***E***, Relative minute ventilation following the injection of saline and fentanyl in control and *Sst-Oprm1*^−/−^ mice. ***F***, Relative minute ventilation averaged over a 5 min period during the early and late phase responses to injection. Data are represented as the mean ± SEM. *p < 0.05. Panels ***A*** and ***B*** were created with BioRender.com.

A systemic injection of fentanyl (0.3 mg/kg; Fujii et al., 2019) was administered and respiratory activity was recorded in control (*Sst*-Cre) and *Sst*-*Oprm1*^−/−^ mice using whole-body plethysmography (Fig. 7*B*,*C*). Systemic fentanyl injection decreased relative minute ventilation compared with saline in both control and *Sst*-*Oprm1*^−/−^ mice (Fig. 7*D*,*E*). During the early phase (minutes 5–10 postinjection), a significant interaction occurred between treatment (saline or fentanyl) and genotype (*p* = 0.0408, two-way repeated-measures ANOVA with Sidak multiple comparisons; Fig. 7*F*). Fentanyl significantly reduced relative minute ventilation compared with saline in both control mice (*p* = 0.0058) and *Sst-Oprm1*^−/−^ mice (*p* < 0.0001). During the late phase of the response (minutes 50–55 postinjection), relative minute ventilation was significantly higher in response to fentanyl compared with saline in *Sst*-Cre mice (*p* = 0.0078, Wilcoxon matched-pairs signed-rank test with Holm–Sidak multiple comparisons) and *Sst-Oprm1*^−/−^ mice (*p* = 0.0391).

**Figure 8.**
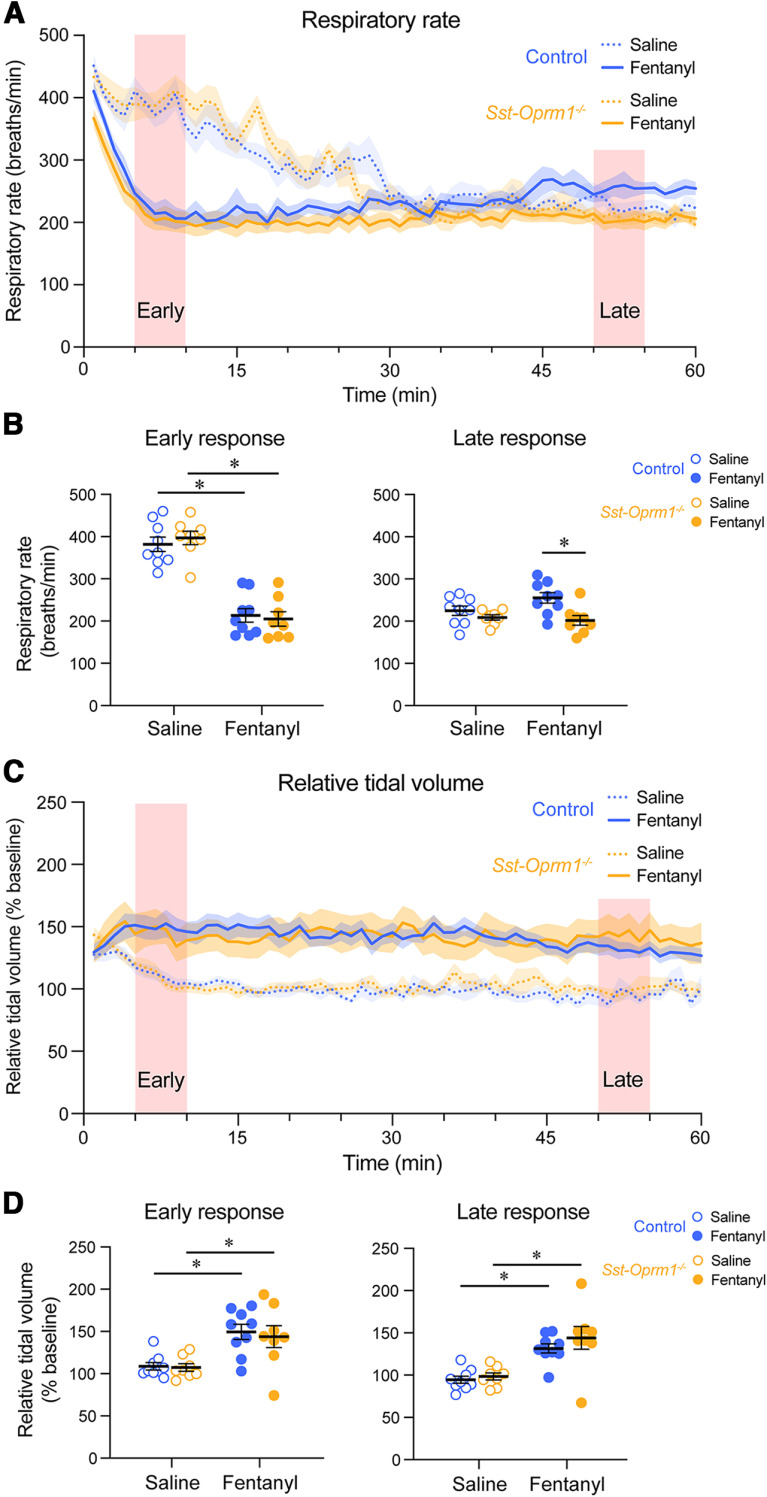
Respiratory rate and relative tidal volume in response to fentanyl in control and *Sst-Oprm1*^−/−^ mice. ***A***, Respiratory rate following the injection of saline or fentanyl in control and *Oprm1*^−/−^ mice. ***B***, Respiratory rate averaged over a 5 min period during the early and late phases of the response to saline and fentanyl. ***C***, Relative tidal volume following the injection of saline or fentanyl. ***D***, Relative tidal volume averaged over a 5 min period during the early and late phases of the response to saline and fentanyl.

Fentanyl depressed respiratory rate in control and *Sst-Oprm1*^−/−^ mice compared with saline (Fig. 8*A*). During the early phase, no significant interaction occurred between treatment (saline or fentanyl) and genotype (*p* = 0.4581, two-way repeated-measures ANOVA with Sidak multiple comparisons; Fig. 8*B*). There was a significant effect of treatment (*p* < 0.0001), where fentanyl induced a significant respiratory rate depression compared with saline injection in both control (*p* < 0.0001) and *Sst-Oprm1*^−/−^ mice (*p* < 0.0001). During the late phase of the response, no significant interaction occurred between treatment (saline or fentanyl) and genotype (*p* = 0.0710, two-way repeated-measures ANOVA with Sidak multiple comparisons), though a significant effect of genotype was found (*p* = 0.0096) with a significant difference between the respiratory rates of control and *Sst*-*Oprm1*^−/−^ mice following fentanyl injection (*p* = 0.0028). No significant differences were found between baseline respiratory rates in control and *Sst*-*Oprm1*^−/−^ mice (*p* = 0.4830, unpaired *t* test with Welch’s correction).

Relative tidal volume was increased in both control and *Sst-Oprm1*^−/−^ mice following fentanyl injection, when compared with saline (Fig. 8*C*). During the early phase, no significant interaction occurred between treatment (saline or fentanyl) and genotype (*p* = 0.7965, two-way repeated-measures ANOVA with Sidak multiple comparisons; Fig. 8*D*). There was a significant effect of treatment (*p* = 0.0002), where fentanyl induced a significant increase in relative tidal volume compared with saline in both control mice (*p* = 0.0043) and *Sst-Oprm1*^−/−^ mice (*p* = 0.0138). During the late phase of the response, no significant interaction occurred between treatment (saline or fentanyl) and genotype (*p* = 0.5354, two-way repeated-measures ANOVA with Sidak multiple comparisons), though a significant effect of treatment was found (*p* < 0.0001) where relative tidal volume was significantly increased in response to fentanyl in control mice (*p* = 0.0023) and *Sst-Oprm1*^−/−^ mice (*p* = 0.0006), compared with saline. Overall, our results show that mice lacking functional MORs in *Sst*-expressing cells presented similar ventilatory depressions compared with control mice following fentanyl administration, suggesting that *Sst*-expressing cells are not involved in respiratory depression by the opioid fentanyl.

### Deletion of MORs in *Sst*-expressing cells and locomotor activity

**Figure 9.**
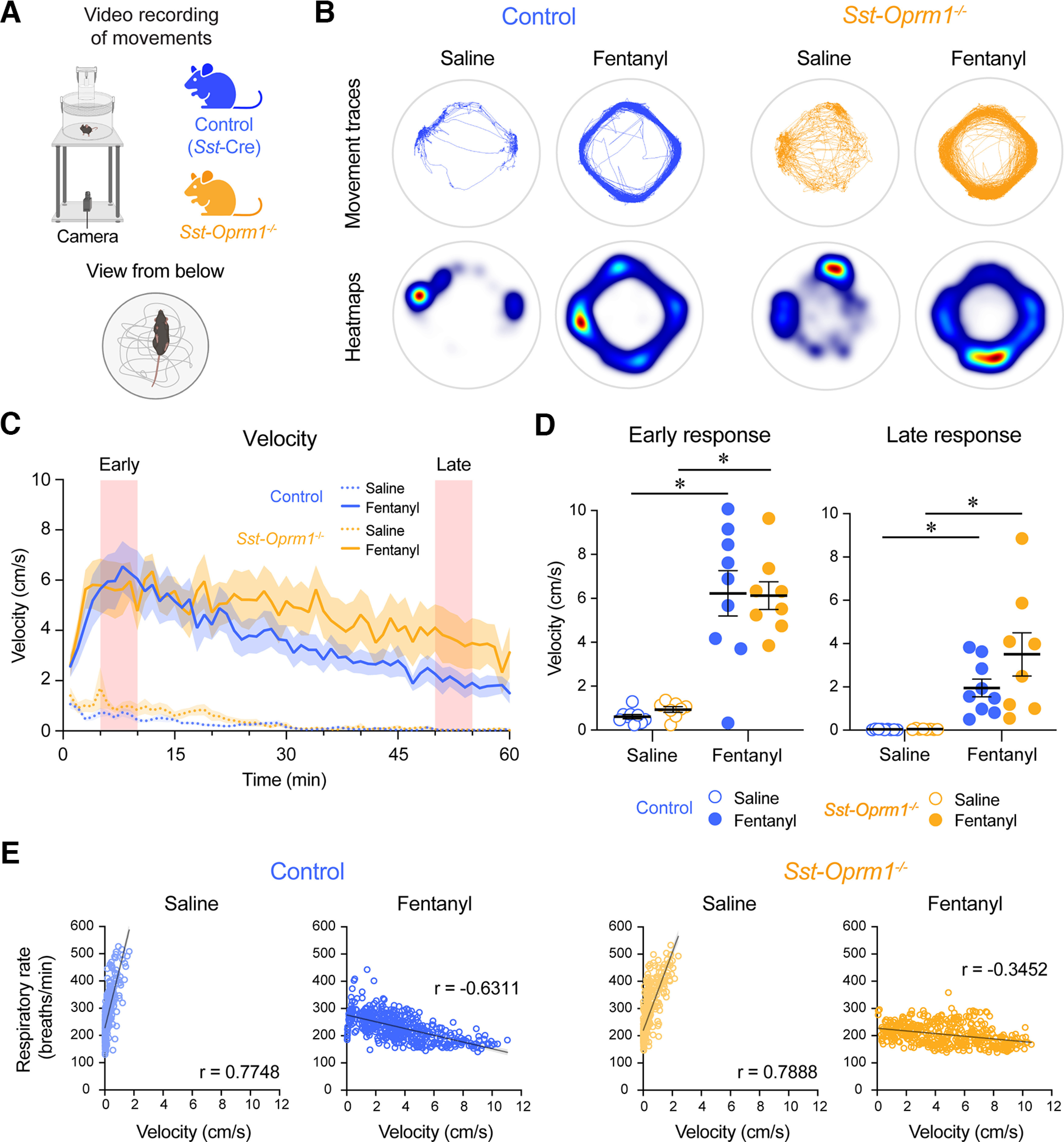
Opioid-induced locomotor hyperactivity in control and *Sst-Oprm1*^−/−^ mice. ***A***, Locomotor activity was recorded using a video camera positioned below the chamber. ***B***, Representative tracings and heat maps of mouse movements during the 1 h postinjection period. ***C***, Average velocity for 1 h after injection of saline or fentanyl in control (*Sst*-Cre) and *Sst-Oprm1*^−/−^ mice. ***D***, Velocity in response to treatment during early and late phases of the response. ***E***, Relationships between velocity and respiratory rate in control and *Sst-Oprm1*^−/−^ mice following saline and fentanyl injection. Data are represented as the mean ± SEM. *p < 0.05. Panel ***A*** was created with BioRender.com.

We examined the locomotor effects of fentanyl in control (*Sst*-Cre) and *Sst*-*Oprm1*^−/−^ mice (Fig. 9*A*). No significant differences were found between baseline velocities of control and *Sst-Oprm1*^−/−^ mice (*p* = 0.0592, Mann–Whitney test). Representative movement traces of control and *Sst*-*Oprm1*^−/−^ mice following injection of either saline or fentanyl are shown in Figure 9*B*. Mouse velocity increased in both control and *Sst*-*Oprm1*^−/−^ mice following fentanyl injection when compared with saline (Fig. 9*C*). The postinjection period was divided into early and late phases (Fig. 9*D*). During the early phase (minutes 5–10 postinjection), no significant interaction occurred between treatment (saline or fentanyl) and genotype (*p* = 0.7501, two-way repeated-measures ANOVA with Sidak multiple comparisons). There was a significant effect of treatment (*p* < 0.0001) where fentanyl significantly increased velocity compared with saline both in control mice (*p* < 0.0001) and *Sst*-*Oprm1*^−/−^ mice (*p* = 0.0001). Similarly, during the late phase (minutes 50–55 postinjection), no significant interaction occurred between treatment (saline or fentanyl) and genotype (*p* = 0.1567, two-way repeated-measures ANOVA with Sidak multiple comparisons). There was, however, a significant effect of treatment (*p* = 0.0001) where velocity was significantly increased in response to fentanyl compared with saline in control mice (*p* = 0.0328) and *Sst*-*Oprm1*^−/−^ mice (*p* = 0.0007). We repeated our analysis examining the relationship between respiratory rate and velocity in control and *Sst*-*Oprm1*^−/−^ mice (Fig. 9*E*). Following saline injection, a significant positive correlation was found between velocity and respiratory rate in both control mice (*r* = 0.7748, *p* < 0.0001) and *Sst*-*Oprm1*^−/−^ mice (*r* = 0.7888, *p* < 0.0001), where increased velocity was associated with increased respiratory rate. Following fentanyl injection, a significant negative correlation was found between velocity and respiratory rate in control mice (*r* = −0.6311, *p* < 0.0001) and a weak negative correlation in *Sst*-*Oprm1*^−/−^ (*r* = −0.3452, *p* < 0.0001), as previously shown in wild-type mice, where greater velocity was associated with lower respiratory rate. Overall, these results show that mice lacking functional MORs in *Sst*-expressing cells and control mice presented with locomotor hyperactivity negatively correlated with the depression of respiratory rate in response to fentanyl.

## Discussion

Opioid drugs have caused countless deaths because of their addictive properties, leading to overdose and severe respiratory depression. Respiratory depression is characterized by slow and shallow breathing that can lead to hypoxemia and death during an overdose (Dahan et al., 2010). Understanding the mechanisms underlying respiratory depression by opioids will ultimately aid in the development of safer opioid pain therapies without potentially lethal side effects. Here, we aimed to identify the circuits mediating the effects of opioid drugs on respiratory rate. Somatostatin-expressing cells are critical for normal breathing (Tan et al., 2008; Cui et al., 2016; de Sousa Abreu et al., 2022) and are found within brainstem respiratory regions shown to contribute to respiratory depression by opioids (Helke, 1984; Johansson et al., 1984; Kalia et al., 1984; De León et al., 1992; Stornetta et al., 2003; Bou Farah et al., 2016; de Sousa Abreu et al., 2022). Importantly, silencing of somatostatin-expressing neurons in key respiratory circuits such as the preBötC, leads to prolonged apneas (Tan et al., 2008); therefore, suggesting that somatostatin-expressing preBötC cells constitute vital components of rhythmic breathing that could contribute to respiratory depression by fentanyl. In our study, we first examined expression of *Sst* and *Oprm1* mRNAs in the brainstem. We found that a majority (>50%) of *Sst* mRNA-expressing cells expressed *Oprm1* mRNA in the preBötC, the NTS, the NA, and the KF nucleus, supporting the hypothesis that these cells may be vulnerable to inhibition by opioids. To determine the role of *Sst*-expressing cells in opioid-induced respiratory depression, we developed conditional knock-out mice that lacked functional MORs in *Sst*-expressing cells. We found that respiratory rate was still depressed by fentanyl in these mice, suggesting that *Sst*-expressing cells are not required for opioid-induced respiratory rate depression.

### *Sst* and *Oprm1* coexpression in brainstem respiratory regions

Both somatostatin and MORs are expressed throughout the brainstem (Johansson et al., 1984; De León et al., 1992; Mansour et al., 1994). Using *in situ* hybridization to quantify *Sst* expression, we found moderate expression of *Sst* mRNAs in the preBötC, the NTS, the NA, and the KF nucleus, with low expression in the ROb and RPa nuclei. Previous studies have found relatively high expression of somatostatin in the preBötC (Stornetta et al., 2003), the NTS (Kalia et al., 1984), and the KF nucleus (Viollet et al., 2008). This difference may be because of our cell counts being expressed as a percentage of total DAPI-stained cells. Since DAPI stains both neuronal and non-neuronal cell nuclei, counts of total DAPI may include non-neuronal cells, which would lower the percentage of cells expressing *Sst*, *Oprm1*, or both. On the other hand, we found high expression of *Oprm1* in the NTS and the NA, moderate expression in the preBötC and the KF nucleus, and low expression in the raphe nuclei. This is consistent with previous studies showing that the NTS (Cerritelli et al., 2016; Zhuang et al., 2017), the NA (Xia and Haddad, 1991), the preBötC (Hayes et al., 2017), and the KF nucleus (Varga et al., 2020b) express MORs. We found that a majority of *Sst* mRNA-expressing cells (>50%) in the preBötC, the NTS, the NA, and the KF nucleus coexpressed *Oprm1* mRNA. Interestingly, these brainstem regions also contribute to opioid-induced respiratory depression. For instance, the preBötC (Montandon et al., 2011; Bachmutsky et al., 2020; Varga et al., 2020a), the NTS (Zhang et al., 2011; Zhuang et al., 2017), the NA (Hassen et al., 1984), and the KF nucleus (Prkic et al., 2012; Levitt et al., 2015; Miller et al., 2017; Saunders and Levitt, 2020) mediate important components of the respiratory side effects of opioids. Our results therefore support the idea that, because of the high expression of *Oprm1* in *Sst* mRNA-expressing cells, these cells may be vulnerable to opioid drugs and contribute to respiratory depression.

### Role of MORs in opioid-induced respiratory rate depression

Most opioid ligands are selective for a wide-range of opioid receptors (Montandon, 2022). For example, fentanyl and morphine bind strongly to the MOR, while morphine also moderately binds to κ-opioid receptors. More specifically, the respiratory depressant effects of fentanyl and morphine are mediated by MORs (Dahan et al., 2001; Hill et al., 2020). Here in our study, we first characterized the respiratory and locomotor response to the opioid fentanyl in wild-type and *Oprm1*^−/−^ mice. During the baseline period, no significant difference in respiratory rate was found between wild-type and *Oprm1*^−/−^ mice. This is in contrast to earlier findings (Dahan et al., 2001) showing a slight increase in respiratory rate in *Oprm1*^−/−^ mice compared with controls. However, this study used hybrids of 129/SV and C57BL/6 mice as controls, whereas we used C57BL/6. We then assessed the response to a relatively high dose of fentanyl (0.3 mg/kg; Fujii et al., 2019) in freely behaving, nonanesthetized wild-type and *Oprm1*^−/−^ mice. Fentanyl decreased relative minute ventilation compared with saline in wild-type mice, but not in *Oprm1*^−/−^ mice, an effect attributed to significant respiratory rate depression following the administration of fentanyl. Fentanyl moderately increased relative tidal volume in wild-type mice, but not in *Oprm1*^−/−^ mice, which is likely because of increased behavioral artifacts collected with plethysmography. In previous studies, fentanyl at this dosage consistently decreased minute ventilation in wild-type mice (Kuo et al., 2015; Varshneya et al., 2022). By contrast, *Oprm1*^−/−^ mice did not show any significant changes in respiratory rate and relative tidal volume following fentanyl injection compared with saline. This is consistent with fentanyl inhibiting neuronal activity by acting on MORs (Montandon, 2022). There were also significant differences in respiratory rates in response to saline during the early postinjection response between wild-type and *Oprm1*^−/−^ mice, despite no differences in baseline breathing rates before injection. It is plausible that the lack of MORs may affect the behavioral and respiratory responses to the stress associated with intraperitoneal injection. In fact, endogenous MOR circuits are involved in stress (Du et al., 2022), and the absence of MORs may affect the stress response following injection. Overall, our results showed that respiratory rate depression by fentanyl was mediated by MORs in freely behaving mice.

### Role of *Sst*-expressing cells in opioid-induced respiratory rate depression

After showing that functional MORs mediate the respiratory rate depressant effects of the opioid fentanyl, we then determined whether MORs in *Sst*-expressing cells contribute to respiratory rate depression. Using a Cre-*loxP* recombination approach, we produced conditional knock-out mice that lacked functional MORs selectively in *Sst*-expressing cells. We compared respiratory responses in control and *Sst-Oprm1*^−/−^ mice. In control mice, fentanyl injection induced a significant decline in respiratory rate compared with saline, as observed in wild-type mice. Surprisingly, fentanyl also decreased respiratory rate and relative minute ventilation in *Sst-Oprm1*^−/−^ mice when compared with saline. Although we found significant differences in respiratory rates between control and *Sst-Oprm1*^−/−^ mice during the late phase following fentanyl injection, the physiological mechanisms mediating these differences are unclear. In addition, fentanyl moderately increased tidal volume in control and *Sst-Oprm1*^−/−^ mice, similar to wild-type mice. Overall, our findings suggest that *Sst*-expressing cells are not involved in opioid-induced rate respiratory depression. These findings are surprising given the coexpression of *Sst* and *Oprm1* mRNAs in regions involved in mediating respiratory depression by opioid drugs and the importance of somatostatin-expressing cells in the modulation of breathing (Tan et al., 2008; Cui et al., 2016; de Sousa Abreu et al., 2022). These results suggest that respiratory depression by fentanyl may involve a population of somatostatin-negative neurons in key medullary regions regulating opioid-induced respiratory depression.

### Opioid-induced locomotor hyperactivity

To ensure that the respiratory effects of opioids were not concealed by movement artifacts in freely behaving mice, we assessed locomotor activity (quantified as velocity) in response to saline and fentanyl. Fentanyl injection induced a significant increase in locomotor activity when compared with saline injection in wild-type, control (*Sst*-Cre), and *Sst-Oprm1*^−/−^ mice, but not in *Oprm1*^−/−^ mice. These findings suggest that while MORs mediate the locomotor effects of opioids, as previously shown (Contarino et al., 2002), the lack of functional MORs in *Sst*-expressing cells does not affect this response. Next, we correlated respiratory rate and velocity to determine whether changes in locomotion may affect respiratory rate. If locomotor activity were a confounding factor for our breathing rate results, we would expect to see a positive correlation between velocity and respiratory rate in response to fentanyl in wild-type mice. Instead, we found a negative correlation in these mice, for which severe respiratory rate depression was correlated with greater locomotor hyperactivity. Interestingly, respiratory rate during opioid-induced respiratory depression and simultaneous locomotor hyperactivity was comparable to respiratory rate during the preinjection baseline where animals were undisturbed for 2 h and velocity was near zero. Our study is the first, to our knowledge, to demonstrate the inverse correlation between the severity of respiratory rate depression and locomotor hyperactivity. In *Oprm1*^−/−^ mice exposed to fentanyl, respiratory rate and locomotor hyperactivity were positively correlated, where increased locomotor activity was associated with increased respiratory rate. Positive correlations were also observed in wild-type mice following saline administration. In both control and *Sst-Oprm1*^−/−^ mice, the severity of respiratory rate depression and locomotor hyperactivity were negatively correlated following fentanyl administration, similar to findings in wild-type mice. This once again shows that the lack of functional MORs in *Sst*-expressing cells did not change the locomotor or respiratory responses to fentanyl. In conclusion, our results demonstrate that, even with increased locomotion following fentanyl administration, respiratory rates were depressed in wild-type, control, and *Sst-Oprm1*^−/−^ mice, but not in *Oprm1*^−/−^ mice.

In summary, we characterized respiratory and locomotor responses to determine the cell types mediating respiratory rate depression by opioids and found that MORs unequivocally mediate the respiratory rate depressive effects of the opioid fentanyl. Considering the role of somatostatin-expressing cells in the control of breathing, we quantified the expression of *Oprm1* mRNA in *Sst*-expressing cells of multiple respiratory circuits involved in mediating the effects of opioids on respiration. We found that a majority (>50%) of *Sst*-expressing cells coexpressed *Oprm1* mRNA in the preBötC, the NTS, the NA, and the KF nucleus. Despite this, the deletion of MORs in *Sst*-expressing cells did not affect respiratory rate depression by fentanyl when compared with control mice. Considering that we measured respiratory rate depression in freely behaving mice by quantifying respiratory activity combined with measures of locomotion, we are confident that respiratory rate depression by fentanyl was accurately assessed. Our results suggest that other cell types may be involved in mediating respiratory rate depression by the opioid fentanyl. MORs are indeed expressed in other cell populations within the brainstem, including cells expressing the neurokinin-1 receptor (Gray et al., 1999). In mice lacking the *Tac1* gene, which encodes the peptide substance P, the endogenous ligand for neurokinin-1 receptors, morphine induced a reduced respiratory depression compared with wild-type mice (Bilkei-Gorzo et al., 2010). In addition, neurokinin-1 receptor-expressing preBötC cells are preferentially inhibited by the MOR agonist DAMGO (Montandon et al., 2011). In conclusion, our study showed that *Sst*-expressing cells are spared from the effects of fentanyl on respiratory rate depression and may constitute a robust neuronal population that can be targeted to stimulate breathing.

## Synthesis

Reviewing Editor: Leandro Vendruscolo, National Institute on Drug Abuse Decisions are customarily a result of the Reviewing Editor and the peer reviewers coming together and discussing their recommendations until a consensus is reached. When revisions are invited, a fact-based synthesis statement explaining their decision and outlining what is needed to prepare a revision will be listed below. The following reviewer(s) agreed to reveal their identity: Stephen Lewis, Renata Marchette.

N/A

## Author Response

We thank the reviewers for carefully reviewing our manuscript. Following their suggestions, we did additional analyses and created new figures. Specifically, we addressed two important points:

1) We calculated the minute ventilation response to fentanyl and saline in all animal groups, as well as tidal volume and included these results in new Figures.

2) We explain our approach to confirm the success of our cre-lox recombination approach to create knockout mice.

Reviewer #1 comments:

This is a terrific study that is only lacking in one key but vital area. Showing frequency of breathing is not adequate to assess the effects of fentanyl since changes in this parameter are closely linked to behavioral and metabolic processes.

I am not doubting your conclusions, but it would seem imperative to include tidal volume data (and minute ventilation data if possible) to establish that your IP dose of fentanyl had actually caused a strong depression of central circuits driving breathing.

RESPONSE. We thank the reviewer for their kind remarks and their consideration of our manuscript. The reviewer raises an excellent point that opioid-induced respiratory depression is characterized by both slow and shallow breathing and in severe cases, inhibition of the hypoxic and hypercapnic ventilatory responses resulting in decreased tidal volume and minute ventilation, the product of respiratory rate and tidal volume (Boland, Boland, & Brooks, 2013; Montandon, 2022). Drorbaugh and Fenn (1955) described a method to record respiratory parameters using plethysmography and to calculate tidal volume from the recorded trace (Drorbaugh & Fenn, 1955). At its core, plethysmography involves detecting pressure changes in the recording chamber that result from inhaled air being warmed up and humidified. From this trace, tidal volume (and by extension minute ventilation) can be calculated provided that accurate and precise measures of core body temperature, chamber humidity, chamber temperature, and atmospheric pressure are acquired during the period being analyzed (Drorbaugh & Fenn, 1955).

In our experiments, we did not measure body temperature, humidity, and chamber temperature because it would require opening the chamber repeatedly and would affect the animal’s behavior, which is an important part of our analysis. Therefore, we calculated tidal volume from the pressure tracings by quantifying the area under the curve during inspiration. We then normalized tidal volume according to its value before injection (baseline) and considered that body temperature, humidity etc. did not change significantly during the measurements. We also calculated relative minute ventilation as the product of tidal volume and respiratory rate. We are now presenting relative minute ventilation, relative tidal volume, and respiratory rate for each experiment in Figures 3, 4, 7, and 8. Fentanyl significantly decreased relative minute ventilation compared to saline, due to substantial reduction of respiratory rate, despite a moderate increase in tidal volume. However, considering the substantial changes in behaviors observed in response to fentanyl, we are cautious when interpretating tidal volume data because it can be affected by behaviors such sniffing. eNeuro, eN-NWR-0035-23 2 2

The following information is now included in Methods.

- To approximate tidal volume, we calculated the area under the curve during inspiration using pressure traces. Since accurate approximation of tidal volume requires measurements of body temperature, chamber humidity and temperature (Mortola and Frappell, 1998), which is challenging and often inaccurate in small rodents (Enhorning et al., 1998), we normalized tidal volume according to baseline values and expressed tidal volume as percentage of baseline, thereby assuming that temperatures and humidity did not change significantly during the experiment. In addition, considering the behavioral changes observed following saline or fentanyl injection, the volume measured with this approach includes behavioral artifacts such as sniffing and movements that may affect the reliability of tidal volume measurements.

- Additionally, we have added the underlined portion below to the following statement in paragraph 1 of the Introduction to clarify differences that can occur in terms of severity of opioid-induced respiratory depression.

- “Through their actions on MORs in respiratory circuits, opioid drugs can cause respiratory depression, characterized by slow and shallow breathing, with severe cases involving inhibition of the hypoxic and hypercapnic ventilatory responses and even that may lead to death with overdose (Dahan et al., 2001; Ramirez et al., 2021).”

Reviewer #2 comments: In their manuscript, the authors present a series of well-designed experiments to assess the role of somatostatin-containing neurons on OIRD. Although the results are very interesting the conclusion that Sst+

-cells do not mediate OIRD, based solely on frequency of breathing, is premature. The control of flow and rhythm parameters of respiration has been proposed to be controlled by different nuclei and cell populations. The authors should also analyze and present flow parameters such as tidal volume, and composite parameters such as minute ventilation and inspiratory drive. Additionally, the presence of apneic events is a hallmark of OIRD, especially with fentanyl, therefore those should be analyzed too.

RESPONSE. We kindly thank the reviewer for their comments and consideration of our manuscript. The reviewer raises an excellent point that the neural network involved in the control of breathing is composed of many regions, each containing diverse subpopulations of neurons that play specific roles in respiratory control (Ramirez et al., 2021). The reviewer also raises the key point that opioids can cause slow and shallow breathing, and in severe cases, inhibition of the hypoxic and hypercapnic ventilatory responses, leading to decline in tidal volume and minute ventilation (the product of tidal volume and respiratory rate) (Boland et al., 2013; Montandon, 2022). We are now including relative tidal volume, i.e. the tidal volume changes in response to saline and fentanyl, expressed as percentage of baseline values before injection. Drorbaugh and Fenn (1955) described a method to record respiratory parameters using plethysmography and to calculate tidal volume from the recorded trace (Drorbaugh & Fenn, 1955). At its core, plethysmography involves detecting pressure changes in the recording eNeuro, eN-NWR-0035-23 3 3 chamber that result from inhaled air being warmed up and humidified. From this trace, tidal volume (and by extension minute ventilation) can be calculated provided that accurate and precise measures of core body temperature, chamber humidity, chamber temperature, and atmospheric pressure are acquired during the period being analyzed (Drorbaugh & Fenn, 1955). In our experiments, we did not measure body temperature, humidity, and chamber temperature because it would require opening the chamber repeatedly and would affect the animal’s behavior, which is an important part of our analysis. Therefore, we calculated tidal volume from the pressure tracings by quantifying the area under the curve during inspiration. We then normalized tidal volume according to its value before injection (baseline) and considered that body temperature, humidity etc. did not change significantly during the measurements. We also calculated relative minute ventilation as the product of tidal volume and respiratory rate. We are now presenting relative minute ventilation, relative tidal volume, and respiratory rate for each experiment in Figures 3, 4, 7, and 8. Fentanyl significantly decreased relative minute ventilation compared to saline, due to substantial reduction of respiratory rate, despite a moderate increase in tidal volume. However, considering the substantial changes in behaviors observed in response to fentanyl, we are cautious when interpretating tidal volume data because it can be affected by behaviors such sniffing.

In addition, we agree with the reviewer that our study does not rule out a potential contribution of Sst-expressing cells to the ventilatory effects of opioids such as fentanyl, considering the changes observed in the late response to fentanyl in SST-Oprm1 knockouts.

RESPONSE. Apneas and opioid-induced respiratory depression. Lastly, the reviewer raises an intriguing point about apneas and opioid-induced respiratory depression. There is an association between central sleep apnea and chronic opioid use (Freire, Sennes, & Polotsky, 2022), where chronic opioid use can lead to central sleep apnea by affecting respiratory rhythm generation in the preBötzinger Complex (Javaheri & Badr, 2023). In the case of obstructive sleep apnea, which involves impaired upper airway function, the presence of this condition can exacerbate opioid-induced respiratory depression (Nagappa, Weingarten, Montandon, Sprung, & Chung, 2017) though there is only a weak association between opioid use and obstructive sleep apnea (Ahmad et al., 2021). In our study, we did not observe any pauses in breathing or central sleep apneas, so we did not find it relevant to look at the effects of fentanyl on apneas.

Minor points:

- in the RNAscope analysis are the Oprm1, Sst, and Oprm1 + Sst three independent populations or are the double-labeled cells part of the first two populations?

RESPONSE. Cells double-labelled for both Oprm1 and Sst mRNAs are part of the first two populations of Oprm1- and Sst-expressing cells.

- the figure showing the validation of the transgenic Sst-Oprm1-/- mice should be included in the supplemental material.

RESPONSE. This is very good point raised by the reviewer. We have added a figure to the

Supplementary Material (Figure 6) describing how our custom primers were used to confirm the excision of exons 2 and 3 from the Oprm1 gene in Sst-Oprm1-/- mice. Primers were designed to eNeuro, eN-NWR-0035-23 4 4 target regions upstream and downstream of the floxed exons 2 and 3 of the Oprm1 gene. The amplicon resulting from this segment is approximately 2980 base pairs long, which is too large for Taq DNA polymerase to amplify, resulting in no band being produced in Sst-Cre and Oprm1fl/fl mice (Arezi, Xing, Sorge, & Hogrefe, 2003; Korbie & Mattick, 2008). With the removal of floxed exons 2 and 3 by Cre, the amplicon is shortened to approximately 500 base pairs and can be amplified by Taq DNA polymerase, producing a mutant band only in Sst-Oprm1-/- that have the floxed segment removed. This approach allows identification of the tissue and animals where the cre-lox recombination occurred. We performed this analysis in control and Sst-Oprm1-/- and in different types of tissues: medulla and earn notch. We found the short amplicon in medulla tissue of Sst-Oprm1-/- mice.

We have also added the underlined segment to the following statement in the Methods section:

“Custom primers were designed and used to confirm knockout of exons 2 and 3 in the Oprm1 gene of Sst-Oprm1-/- mice using touchdown PCR (Figure 6) (Korbie & Mattick, 2008). Briefly, the Oprm1 gene in Oprm1fl/fl mice was floxed in regions upstream and downstream of exons 2 and 3. Primers were designed to target these floxed exon-flanking regions. Without the removal of exons 2 and 3 by Cre, the entire segment would be too large to amplify using Taq DNA polymerase and no band was produced in Sst-Cre or Oprm1fl/fl mice (Arezi et al., 2003). After removal of exons 2 and 3 by Cre, as occurred in Sst-Oprm1-/- mice, the primer-targeted regions were in closer proximity to each other and the fragment was amplified and a mutant band was produced indicating the deletion.” - the injection volume is not stated.

RESPONSE. The volume of saline or fentanyl (0.3mg/kg) (Fujii, Koshidaka, Adachi, & Takao, 2019) injected was determined based on mouse weight on each experimental day. We calculated that to achieve a dose of 0.3mg/kg of fentanyl by intraperitoneal injection, a volume of 120μL is needed per 20g mouse. Injected volumes ranged between 120-240μL, depending on mouse weight. We have added the following statement to the Methods section, under Whole-Body

Plethysmography in the revised manuscript:

“The volume injected to achieve a dose of 0.3mg/kg of fentanyl was calculated to be 120μL per 20g mouse. The injected volumes of saline and fentanyl were calculated on each respective experimental day based on the mouse’s weight taken at 10:00 and ranged between 120-240μL, depending on mouse weight.”
